# P-72. 28-Day All-cause Mortality and Associated Factors in Cancer Patients with Bacteremia in a Peruvian Referral Center

**DOI:** 10.1093/ofid/ofaf695.301

**Published:** 2026-01-11

**Authors:** Yosué I Vera, Nicolás Zamudio, P E D R O E LEGUA, Dany J RIVERA CRUZADO, Kathiuska Z Tutaya Chávez, Yenka M La Rosa, Paola Montenegro, Ivan C Aguilar, Karol M Villavicencio, Angie Y Palomino, Frank Young, Carlos Seas

**Affiliations:** Clinica Oncosalud, Lima, Lima, Peru; Universidad Peruana Cayetano Heredia, Lima, Lima, Peru; Instituto de Medicina Tropical "Alexander von Humboldt" - Universidad Peruana Cayetano Heredia, LIMA, Lima, Peru; Laboratorio de Microbiología, AUNA, SANTIAGO DE SURCO, Lima, Peru; Laboratorio de Microbiologia, AUNA, lima, Lima, Peru; Clínica Oncosalud, Lima, Lima, Peru; Clínica Oncosalud, Lima, Lima, Peru; Clínica Oncosalud, Lima, Lima, Peru; Clínica Oncosalud, Lima, Lima, Peru; Clínica Oncosalud, Lima, Lima, Peru; Clínica Oncosalud, Lima, Lima, Peru; Instituto de Medicina Tropical Alexander von Humboldt - UPCH, Lima, Lima, Peru

## Abstract

**Background:**

The global prevalence of cancer has increased in recent decades. Cancer patients are particularly susceptible to invasive infections such as bacteremia. In Peru, the rising incidence of bloodstream infections caused by resistant pathogens represents a significant public health concern. However, limited information is available on this complication in the Peruvian oncologic population. No previous studies in the country have assessed mortality or examined the impact of antimicrobial resistance on patient outcomes in this population.

**Figure:**
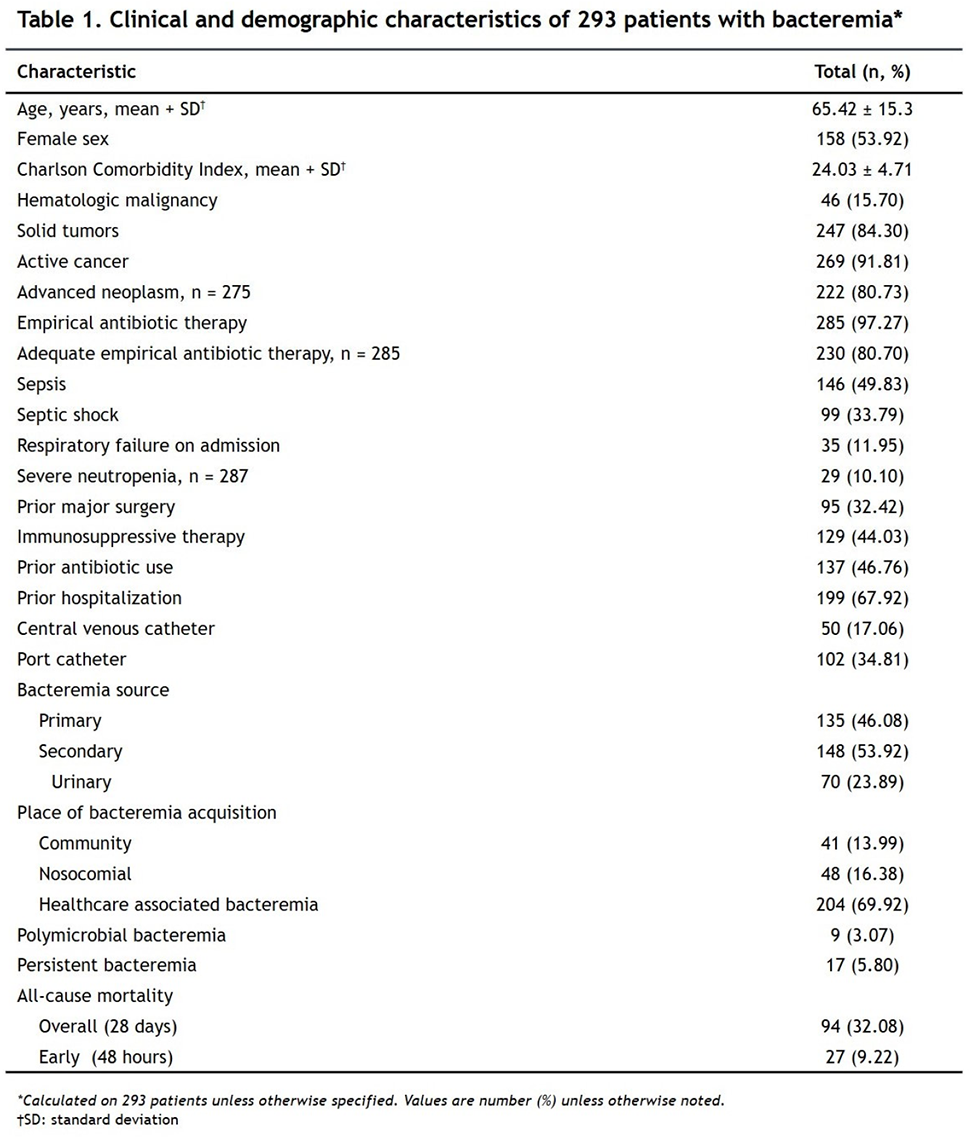

**Figure:**
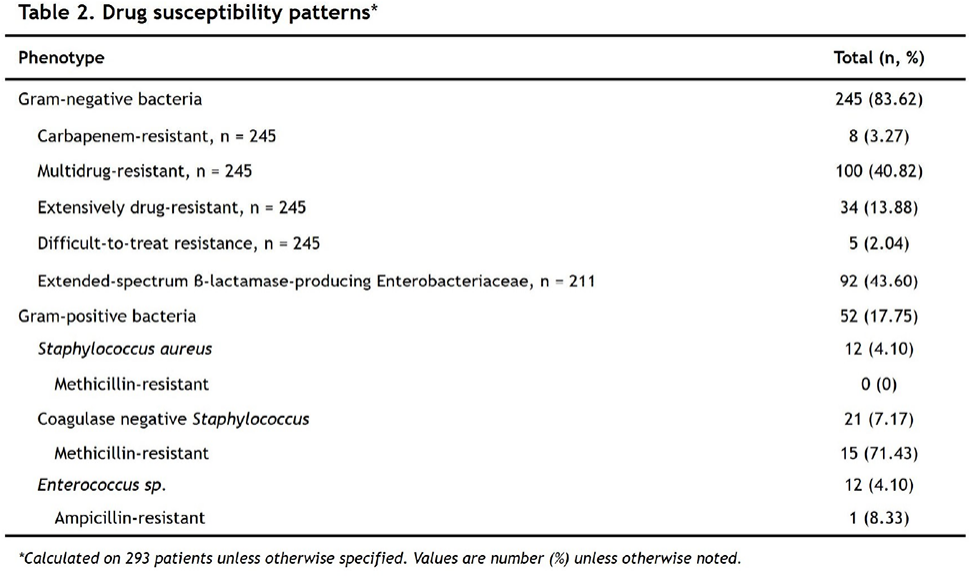

**Methods:**

This study aimed to evaluate 28-day all-cause mortality and its associated factors among cancer patients with bacteremia at a referral cancer center in Lima. We retrospectively analyzed data from first episodes of bacteremia in hospitalized adult patients between July 2020 and June 2024.

**Figure:**
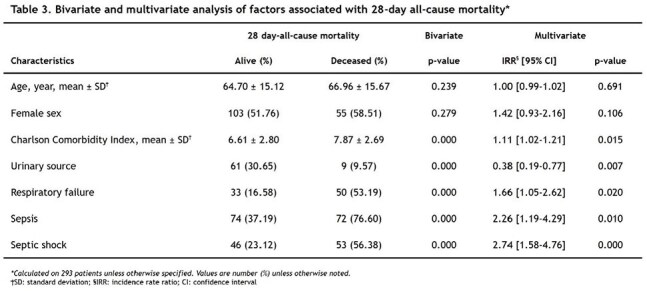

**Figure:**
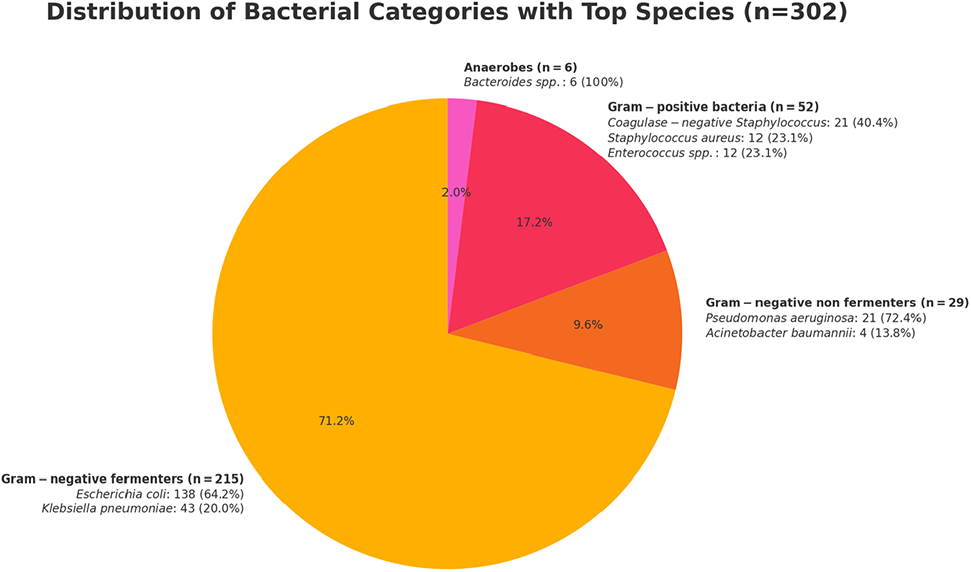

**Results:**

A total of 293 patients were included. The mean age was 65.42 ± 15.31 years, and 53.92% were female. Most patients had solid tumors (84.30%) and active disease (91.81%), with digestive cancers being the most common (34.13%). The 28-day all-cause mortality rate was 32.08%. Empirical antimicrobial therapy was appropriate in 80.70% of cases, Table 1. Gram-negative bacteria predominated (82.80%), with *Escherichia coli* being the most frequently isolated pathogen (45.70%). Among enterobacteria, 43.60% were ESBL producers, and 3.27% of gram-negative isolates were carbapenem-resistant, Figure 1 and Table 2. Multivariate Poisson regression identified the Charlson Comorbidity Index (IRR 1.11; 95% CI 1.02–1.21), sepsis (IRR 2.26; 95% CI 1.19–4.29), septic shock (IRR 2.74; 95% CI 1.58–4.76), and respiratory failure (IRR 1.66; 95% CI 1.05–2.62) as independent factors associated with increased 28-day mortality. A urinary source of infection was found to be protective (IRR 0.38; 95% CI 0.19–0.77), Table 3.

**Conclusion:**

In this cancer referral center, one-third of patients with bacteremia died within 28 days. Mortality was primarily driven by infection severity and specific comorbidities rather than antimicrobial resistance.

**Disclosures:**

All Authors: No reported disclosures

